# Initial investigation on the feasibility of porcine red blood cells from genetically modified pigs as an alternative to human red blood cells for transfusion

**DOI:** 10.3389/fimmu.2023.1298035

**Published:** 2023-11-14

**Authors:** Sangkeun Park, Haneulnari Lee, Eun Mi Park, Juhye Roh, Pul Ip Kang, Joohyun Shim, Kimyung Choi, Hee Jung Kang

**Affiliations:** ^1^ Department of Laboratory Medicine, Hallym University Sacred Heart Hospital, Anyang, Republic of Korea; ^2^ Department of Laboratory Medicine, Hallym University College of Medicine, Chuncheon, Republic of Korea; ^3^ Department of Transgenic Animal Research, Optipharm Inc., Cheongju, Republic of Korea

**Keywords:** genetically modified pigs, red blood cells, transfusion, hemolysis, complement activation, CD55

## Abstract

The decline in blood donation rates and the ongoing shortage of blood products pose significant challenges to medical societies. One potential solution is to use porcine red blood cells (pRBCs) from genetically modified pigs as an alternative to human red blood cells (hRBCs). However, adverse immunological reactions remain a significant obstacle to their use. This study aimed to evaluate the compatibility of diverse genetically modified pRBCs with human serum. We acquired human complement-competent serum, complement 7 (C7)-deficient serum, and hRBCs from all ABO blood types. Additionally, we used leftover clinical samples from health checkups for further evaluation. pRBCs were collected from wild-type (WT) and genetically modified pigs: triple knockout (TKO), quadruple KO (QKO), and TKO/hCD55.hCD39 knockin (hCD55.hCD39KI). The extent of C3 deposition on RBCs was measured using flow cytometry after incubation in C7-deficient serum diluted in Ca^++^-enriched or Ca^++^-depleted and Mg^++^-enriched buffers. The binding of immunoglobulin (Ig) M/IgG antibody to RBCs after incubation in ABO-type human serum was evaluated using flow cytometry. Naïve human serum- or sensitized monkey serum-mediated hemolysis was also evaluated. Phagocytosis was assessed by incubating labeled RBCs with the human monocytic cell line THP-1 and measurement by flow cytometry. All three genetic modifications significantly improved the compatibility of pRBCs with human serum relative to that of WT pRBCs. The extent of IgM/IgG binding to genetically modified pRBCs was lower than that of WT pRBCs and similar to that of O-type hRBCs. Total and alternative pathway complement activation in all three genetically modified pRBCs was significantly weaker than that in WT pRBCs and did not differ from that in O-type hRBCs. The extent of serum-mediated hemolysis and phagocytosis of these genetically modified pRBCs was low and similar to that of O-type hRBCs. Sensitized monkey serum-mediated hemolysis in QKO and TKO/hCD55.hCD39KI pRBCs was higher than in O-type hRBCs but lower than in TKO pRBCs. The elimination of porcine carbohydrate antigens in genetically modified pigs significantly enhanced pRBC compatibility with naïve human sera, which was comparable to that of O-type hRBCs. These findings provide valuable insights into the development of pRBCs as potential alternatives to hRBCs.

## 1 Introduction

The steady decline in blood donation rates and recurring shortages of blood products raise concerns within medical societies (1). The increasing fear of infectious disease transmission restricts donor eligibility and raises the rate of donor exclusion, such that the limited supply of blood products cannot be easily resolved. Several approaches for developing an alternative replacement for human red blood cell (hRBC) transfusion include hemoglobin derivatives and stem cell-based therapy, which have had little clinical success (2, 3). Due to advances in genetic engineering technologies (4, 5), porcine RBCs (pRBCs) from genetically modified pigs have been investigated as alternatives to hRBCs for transfusion (6, 7). The supply of pRBCs would be unlimited, and the risk of infection transmission could be more easily controlled compared to human blood. However, to bring this technology into clinical practice, a few challenges need to be addressed, including immunological and physiological barriers, the potential risk of xenozoonosis, and ethical concerns about using animals for human purposes (8–11). Specifically, immunological barriers, such as intravascular and/or extravascular hemolysis following pRBC transfusion, represent the initial obstacles on the road to clinical xenotransfusion (6).

Humans naturally acquire antibodies against several porcine carbohydrate antigens that share antigenicity with environmental microbes and food (12, 13). Galactose-α1,3-galactose (Gal) and, to a lesser extent, *N*-glycolylneuraminic acid (Neu5Gc) and Sd(a) are expressed on the surface of porcine cells, and humans naturally acquire antibodies against them (14). Deletion of Gal in donor pigs by *alpha-1,3-galactosyltransferase* gene-knockout (*GT*KO) genetic modification reduces antibody binding and subsequent hemolysis of pRBCs after incubation with naïve human sera (15). However, compared with hRBCs in ABO-compatible human sera, *GT*KO pRBCs in human sera show higher antibody-mediated hemolysis and phagocytosis by human monocyte-derived phagocytes (16). Additional deletion of the Neu5Gc antigen in donor pigs by targeting the *cytidine-5′-monophosphate-N-acetylneuraminic acid hydroxylase* (*CMAH*) gene further reduces antibody-mediated hemolysis in human sera (17), as does further depleting the Sd(a) antigen by targeting the *β-1,4-N-acetylgalactosaminyltransferase 2* (*B4GalNT2*) gene. Accordingly, triple knockout (TKO) gene modification achieved the lowest antibody binding to porcine cells in human sera (5, 18). Yamamoto et al. demonstrated the *in vivo* survival of TKO pRBCs for several days in the circulation of capuchin monkeys (*Cebus*) and suggested the possibility of using TKO pRBCs as an alternative to hRBCs (19).

In our previous study (20), anti-Gal and anti-Neu5Gc IgG and IgM antibodies were detected in most healthy Koreans, supporting the necessity of *GT*KO and *CMAH*KO gene modifications in donor pigs. Incidentally, altering sialic acid composition by *CMAH*KO gene modification does not hamper factor H-mediated complement inhibition in porcine cells incubated in human serum (21). When incubated in human platelet-rich plasma, TKO porcine cells showed delayed platelet aggregation compared with that in wild-type (WT) or *GT*KO/*CMAH*KO porcine cells (21). These results suggest that TKO genetic modification in donor pigs is a baseline platform for developing alternatives to hRBCs. Recently, Ko et al. (22) reported the development of multigene-modified pigs that express human CD55 and CD39 on their RBCs and other tissues in addition to deletions of Gal and Neu5Gc. CD55 expressed on RBCs plays an important role in protecting RBCs from complement-mediated attacks (23). Thus, human CD55 expression may confer further protection to TKO pRBCs against human complement-mediated lysis (24). Additionally, there was a concern that *GT*KO gene modification might not be sufficient to completely eliminate the Gal epitope (25). However, Butler et al. subsequently reported that silencing the porcine *A3GalT2* gene in *GT*KO pigs alters the renal sphingolipid profile. These alterations did not affect Gal levels on *GT*KO pig cells or change the cross-match cytotoxicity of the peripheral blood mononuclear cells from *GT*KO and *GT*KO/*A3GalT2*KO pigs when exposed to naïve baboon and human sera (26). The residual expression of the Gal epitope on glycosphingolipids is not likely to contribute to anti-Gal antibody-related cytotoxicity, but silencing the *A3GalT2* gene might lead to alterations in glycosphingolipid or other antigen profiles on the membrane of pRBCs, which might affect compatibility tests of *A3GalT2*KO pRBCs in human sera.

In this study, we aimed to determine the best candidates as alternatives to hRBCs. Therefore, we investigated the additive effect of further genetic modifications to TKO modification on the compatibility of pRBCs in human serum. We evaluated the performance of various pRBCs from WT, TKO, quadruple (*GT*/*CMAH/B4GalNT2/A3GalT2*) knockout (QKO), and TKO plus human CD55 and CD39 knockin (TKO/hCD55.hCD39KI) pigs relative to that of ABO-compatible and -incompatible hRBCs in terms of antibody reactivity, complement deposition, hemolysis in human serum, and phagocytosis by human monocytes.

## 2 Materials and methods

### 2.1 Cells and human or monkey serum

RBCs from WT and genetically modified pigs were provided by Optipharm Inc. (Cheongju, Korea): WT, TKO, TKO/hCD55.hCD39KI, and QKO pRBCs (**Table 1**). AB- and O-type hRBCs were purchased from Bio-Rad Laboratories (Hercules, CA, USA), and complement-competent sera and C7-depleted sera were purchased from Quidel Corp. (San Diego, CA, USA). Leftover clinical samples from health checkups were obtained and used for further validation. The institutional review board of Hallym University Sacred Heart Hospital approved the study design (HALLYM 2022-12-002). Sensitized monkey serum was obtained from a porcine xenotransplanted cynomolgus monkey with a high titer of anti-pig cell antibodies (27). The human monocytic leukemia cell line THP-1 (ATCC, Manassas, VA, USA) was used to assess phagocytosis (28).

**Table 1:** Details of the genetically modified pigs used in this study.

Type of pig	Details of genetic modification
Triple knockout (TKO)	*1,3-galactosyltransferase* gene-, *cytidine monophosphate-N-acetylneuraminic acid hydroxylase* gene-, and *β1,4 N-acetylgalactosaminyltransferase 2* gene-knockout
Quadruple knockout (QKO)	TKO and *isoglobotrihexosylceramide synthase* gene-knockout
TKO/hCD55.hCD39KI	TKO and human *CD55* gene- and human *CD39* gene-knockin

### 2.2 Assessment of complement activation on RBCs by C7-depleted serum

Two million RBCs in single-cell suspensions were incubated in 100 µL of 0, 10, 20, and 30% human C7-depleted serum diluted in Ca^++^- and Mg^++^-enriched gelatin veronal buffer (GVB^++^, for total complement activation) or Mg^++^-EGTA-GVB (for alternative pathway complement activation) at 37°C for 30 min and then stained with fluorescein-conjugated goat IgG fraction to human complement C3 (MP Biomedicals, Solon, OH, USA) (21, 29–31). C7-depleted serum allows complement activation of RBCs but prevents complement-mediated lysis of RBCs during the assays. The RBCs were analyzed using a Cytoflex flow cytometer (Beckman Coulter, Brea, CA, USA). The amount of C3 deposited on the RBCs was expressed as the net mean fluorescence intensity (MFI) by subtracting the MFI of the sample without human serum (0%) from the MFI of the sample with human serum.

### 2.3 Assessment of antibody binding to RBCs by human serum

Each 2 × 10^5^ RBC suspension was incubated in 50 µL of 10% human serum diluted in phosphate-buffered saline (PBS) containing 1% bovine serum albumin (BSA) and 33 mM EDTA at 37°C for 30 min and then stained with fluorescein-conjugated F(ab)2 fragments of rabbit immunoglobulins specific for human IgG or IgM (Dako, Santa Clara, CA, USA). The incubation temperature was chosen by referring to the protocols of antibody screening tests in clinical laboratories or blood banks (32). The RBCs were analyzed using a Cytoflex flow cytometer (Beckman Coulter). The amount of antibody bound to RBCs was expressed as the MFI.

### 2.4 Assessment of human serum-mediated hemolysis

To assess human serum-mediated hemolysis, 2 × 10^6^ RBCs were suspended in 100 µL of 50% human serum diluted in GVB^++^ or GVB-containing 10 mM EDTA (GVB-EDTA) and incubated at 37°C for 30 min. After centrifugation, 90 µL of supernatant was transferred into an ELISA plate well, and the absorbance at 412 nm (Abs) was measured using an Epoch ELISA reader (BioTek Instruments Inc., Winooski, VT, USA). The suspension of the same number of the RBCs alone was incubated in 100 µL of GVB^++^ to validate the intactness of the RBCs and in 100 µL distilled water (DW) as a 100% hemolysis control in parallel. Each sample was tested in duplicate. The percentage hemolysis was calculated as follows:


$$\frac{\left(Abs\;of\;the\;supernatant_{serum\;in\;GVB++}-Abs\;of\;the\;supernatant_{serum\;in\;GVB-EDTA}\right)}{\left(Abs\;of\;the\;supernatant_{DW}-Abs\;of\;DW\right)}\;\times 100$$

The assays were considered valid only when the hemolysis of RBCs in 100 µL of GVB^++^ was less than 5%.

### 2.5 Assessment of phagocytosis

The RBCs (5 × 10^7^) were suspended in PBS and labeled with carboxyfluorescein diacetate succinimidyl ester (CFSE; Invitrogen, Carlsbad, CA, USA) at a final concentration of 5 μmol/L for 10 min at 37°C. The stained RBCs were washed twice and resuspended in complete RPMI medium. The stained RBCs (4 × 10^5^) and THP-1 cells (1 × 10^5^) were mixed in 100 μL RPMI complete media with 10% fetal bovine serum (FBS) or 10% AB-type human serum and incubated for 3 h at 37°C. Thereafter, the cells were washed and treated with BD FACS™ Lysing Solution (BD Biosciences, Franklin Lakes, NJ, USA) to remove the remaining unphagocytosed RBCs. The extent of phagocytosis of CFSE-labeled RBCs by THP-1 cells was assayed using flow cytometry and expressed as the MFI.

### 2.6 Statistical analysis

Data are expressed as the mean ± standard deviation of at least three independent experiments in duplicate. Differences between pairs of groups were compared using the Kruskal–Wallis or Wilcoxon test. Statistical significance was set at *P*< 0.05.

## 3 Results

### 3.1 Pathway-specific complement activation on pRBCs

**Figure 1:**
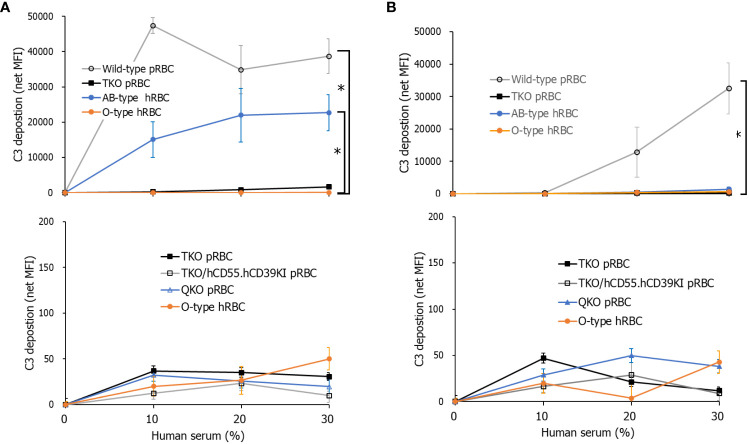
Total and alternative pathway complement activation on porcine red blood cells (pRBCs) or human RBCs (hRBCs) incubated in C7-depleted human serum. Various wild-type (WT) or genetically modified pRBCs and AB- or O-type hRBCs were incubated in C7-depleted human serum diluted in Ca^++^ enriched gelatin veronal buffer (GVB^++^) **(A)** or Mg^++^-EGTA-GVB **(B)** at 37°C; they were then stained with anti-C3 antibodies. C3 deposition on the RBCs was analyzed by flow cytometry. The experiment was replicated at least three times in duplicate. *P< 0.05, Kruskal–Wallis test. RBCs, red blood cells.

When we assessed the deposition of C3 on RBCs in C7-depleted human serum diluted in GVB^++^, the amount of C3 deposition on WT pRBCs and AB-type hRBCs increased with increasing human serum concentrations, but that on TKO pRBCs and O-type hRBCs did not (**Figure 1A**). The C3 net MFI values of TKO pRBCs and O-type hRBCs were significantly lower than those of WT pRBCs in 30% human serum (*P* = 0.0139). The C3 net MFI values did not differ among TKO pRBCs, QKO pRBCs, TKO/hCD55.hCD59KI pRBCs, and O-type hRBCs. Meanwhile, when we evaluated alternative pathway-specific complement activation using Mg^++^-EGTA-GVB conditions, only WT pRBCs showed increased C3 deposition with increasing human serum concentrations, while the others did not (**Figure 1B**). The C3 net MFI values of WT pRBCs in 30% human serum in Mg^++^-EGTA-GVB were significantly higher than those of TKO pRBCs, O-type hRBCs, and AB-type hRBCs (*P* = 0.0204). The C3 net MFI values of TKO pRBCs, TKO/hCD55.hCD59KI pRBCs, QKO pRBCs, and O-type hRBCs did not differ. Notably, C3 deposition on AB-type hRBCs was substantial in human serum diluted in GVB^++^ (**Figure 1A**) but rarely observed in human serum diluted in Mg^++^-EGTA-GVB (**Figure 1B**).

### 3.2 Antibody binding to pRBCs incubated in human sera

**Figure 2:**
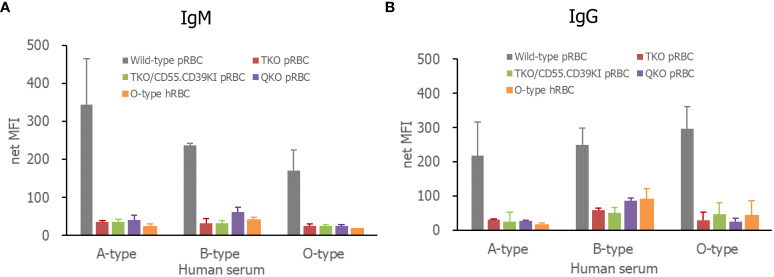
Human antibody binding to pRBCs or hRBCs, IgM **(A)**, and IgG **(B)**. The RBCs were incubated in various ABO-type 10% human serum in PBS containing 1% bovine serum albumin and 33 mM EDTA. Bound antibodies were detected by fluorescein-conjugated F(ab)2 fragments of a rabbit immunoglobulin specific for human IgG or IgM and analyzed by flow cytometry. RBCs, red blood cells; PBS, phosphate-buffered saline.

When each RBC suspension was incubated with human sera of various ABO types, human IgG or IgM in all sera, including A-, B-, and O-types, bound to WT pRBCs to a greater extent than to TKO, QKO, and TKO/hCD55.hCD59KI pRBCs or O-type hRBCs (**Figure 2**). The IgG and IgM MFI values for these genetically modified pRBCs and O-type hRBCs did not differ, although there were insignificant variations in the values depending on the test serum.

### 3.3 Assessment of human serum-mediated hemolysis

**Figure 3:**
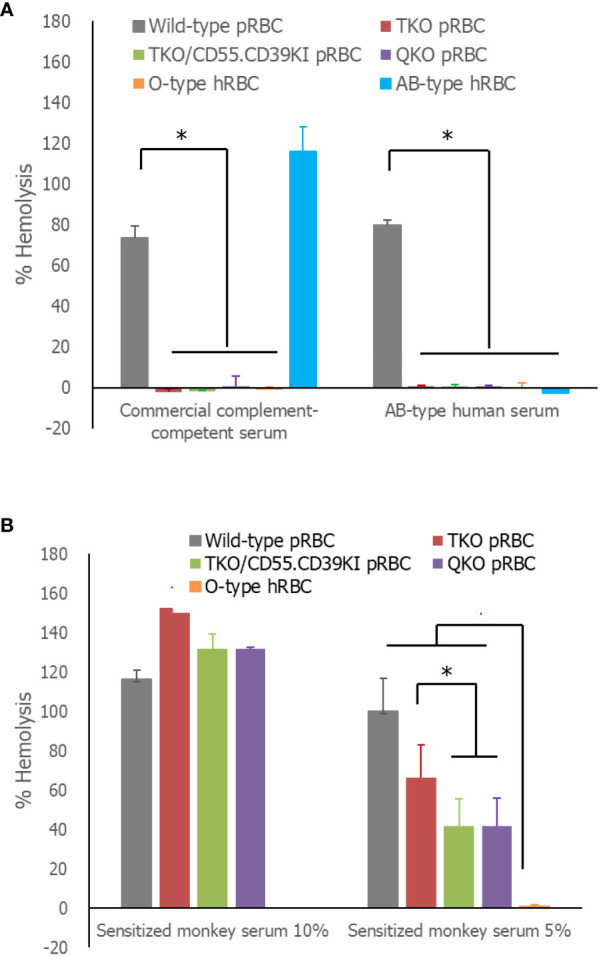
Serum-mediated hemolysis of RBCs. **(A)** The RBCs were incubated in 50% human serum diluted in Ca^++^-enriched gelatin veronal buffer (GVB^++^) or GVB-EDTA, and the absorbance of the supernatants at 412 nm was measured. The absorbance following hemolysis of the sample was determined as the difference of absorbances between GVB^++^ and GVB-EDTA samples. The % hemolysis of the sample was calculated from the absorbance of the complete hemolysis tube (100%). **(B)** The same experiment was performed using 10 or 5% previously sensitized monkey serum instead of human serum. Each experiment was replicated at least three times in duplicate. *P< 0.05, Wilcoxon paired test. RBCs, red blood cells.

In the comparison of the extent of lysis of RBCs by human serum, more than 70% of WT pRBCs and AB-type hRBCs were lysed in complement-competent human serum, whereas the extent of hemolysis of the three genetically modified pRBCs and O-type hRBCs was less than 10%, significantly lower than that of WT pRBCs (**Figure 3A**). In AB-type human serum, hemolysis of WT pRBCs was over 70%, whereas the other RBCs, including AB-type hRBCs, were rarely hemolyzed. Nevertheless, the extent of hemolysis was insufficient to determine the effect of hCD55 function on TKO/hCD55.hCD39KI pRBCs. We used sensitized monkey serum to enhance the hemolysis of pRBCs. All pRBCs, regardless of the genetic modification, were completely hemolyzed in 10% sensitized monkey serum, whereas O-type hRBCs were minimally hemolyzed (**Figure 3B**). When we repeated the same experiment with 5% serum, WT pRBCs were completely hemolyzed; however, genetically modified pRBCs were partially protected from hemolysis at different levels (**Figure 3B**). The percentage hemolysis of TKO/hCD55.hCD59KI pRBCs and QKO pRBCs was significantly lower than that of TKO pRBCs (*P* = 0.0059 and *P* = 0.0273, respectively; **Figure 3B**). O-type hRBCs were rarely hemolyzed, and their % hemolysis was significantly lower than that of all pRBC types (*P* = 0.0002).

### 3.4 Assessment of phagocytosis

**Figure 4:**
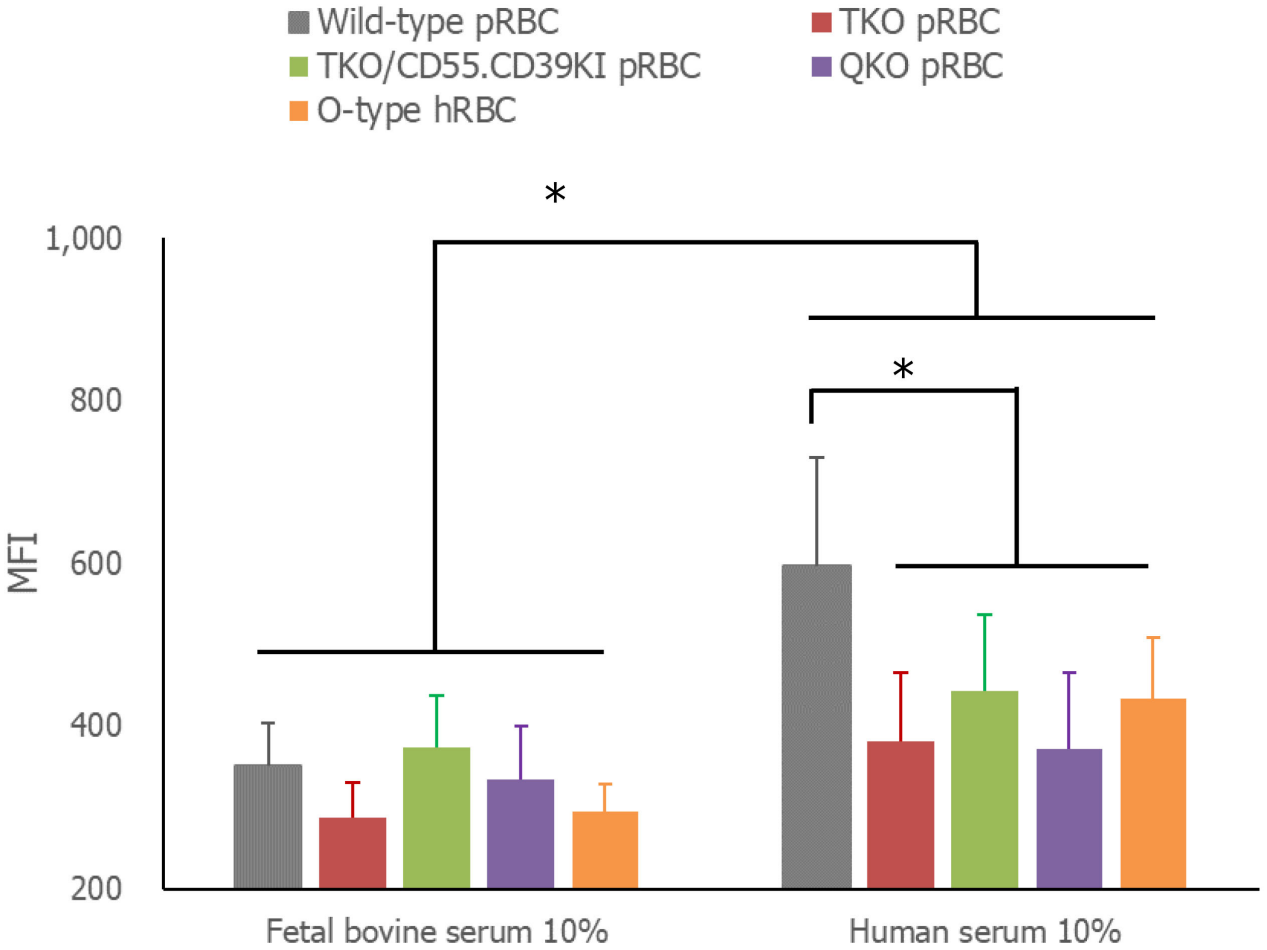
Phagocytosis of RBCs by THP-1 cells. The RBCs were labeled with carboxyfluorescein diacetate succinimidyl ester (CFSE) and incubated with THP-1 cells in RPMI complete media with 10% fetal bovine serum or 10% AB-type human serum for 3 h at 37°C. Unphagocytosed RBCs were removed by treatment with BD FACS™ Lysing solution. The extent of CFSE-labeled RBC phagocytosis by THP-1 cells was measured by flow cytometry. *P< 0.05, Wilcoxon paired test. RBCs, red blood cells.

In the comparison of the extent of RBC phagocytosis by human monocytes, the amount of RBCs phagocytosed by THP-1 cells in media containing 10% FBS did not differ among the tested RBCs: WT, TKO, TKO/hCD55.hCD59KI, QKO pRBCs, and O-type hRBCs (**Figure 4**). However, phagocytosis was significantly enhanced in media containing human serum compared with that in FBS-containing media for each type of RBC (*P<* 0.001). Moreover, the phagocytosis of WT pRBCs was facilitated by human serum-containing media, and the MFI value of up-taken WT pRBCs was significantly higher than those of other RBCs in the same human serum-containing media (*P* = 0.0387). The MFI values of the phagocytosed genetically modified pRBCs did not differ from those of O-type hRBCs.

## 4 Discussion

Blood product shortages due to a decreasing supply are a potential concern for the medical community. It is necessary to develop a new blood substitute, and animal blood, including that of pigs, may have potential as an alternative to hRBCs (33). Some glycans on pig cells are shared with ubiquitous microbes but not with primates, including humans; therefore, humans naturally acquire antibodies against them (20, 34). WT pRBCs undergo serum-mediated lysis immediately after contact with human or nonhuman primate blood. However, genetic modification of donor pigs can improve the compatibility of pRBCs with human blood (6). Previous studies have reported that *GT*KO pRBCs are less agglutinated in human serum than WT pRBCs or ABO-incompatible hRBCs, although not comparable to ABO-compatible hRBCs (16). Additional deletion of *CMAH* from donor pigs further reduces human antibody binding to pRBCs (17). Recently, *GT*/*CMAH/B4GalNT2* TKO modification achieved the lowest antibody binding to porcine cells in human sera (18), and TKO pRBCs survived for several days in the circulation of capuchin monkeys (19). However, these improvements were insufficient for the use of pRBCs as a substitute for hRBCs, and further modifications are required to improve their compatibility. In this study, we explored the *in vitro* compatibility of pRBCs with several genetic modifications, including TKO, QKO, and TKO/hCD55.hCD39KI. Surprisingly, these pRBCs exhibited excellent compatibility with human serum, comparable to that of O-type human RBCs, which are considered universal donor RBCs. Moreover, hCD55 expression conferred greater protection against antibody-mediated hemolysis in TKO pRBCs. These results suggest that pRBCs could be developed as alternatives to hRBCs.

Because normal human serum easily lyses incompatible RBCs, the assessment of C3 deposition on RBCs in normal serum using flow cytometry is inaccurate. To circumvent this, we used commercial C7-depleted human serum to evaluate complement activation on RBCs. Under Ca^++^-enriched conditions, increases in C3 deposition along with increases in the concentration of C7-depleted human serum were found on WT pRBCs and AB-type hRBCs, whereas no increase in C3 deposition on all three genetically modified pRBCs relative to that on O-type hRBCs was observed. The ABO type of this commercial serum was not provided; however, we presumed that AB-type hRBCs would be ABO-incompatible with this serum. Meanwhile, WT pig cells have been shown to activate complement *via* the alternate pathway (21, 31). Accordingly, under Ca^++^-depleted conditions, which allow only alternative pathway activation, a significant increase in C3 deposition was found only on WT pRBCs and not on genetically modified pRBCs, suggesting that the surface of these genetically modified pRBCs is not prone to the activation of the human complement system, similar to hRBCs.

We compared the binding of IgG and IgM antibodies to pRBCs in different ABO-type naïve human sera. WT pRBCs showed high antibody binding in all tested sera, whereas none of the genetically modified pRBCs or O-type hRBCs showed significant antibody binding in different ABO sera, except for minor individual variations. These results are in line with previous reports demonstrating that TKO gene modification reduces the levels of human antibody binding to pRBCs, which is as low as autologous hRBCs (5). In addition, our results for human serum-mediated hemolysis agree well with these antibody results. Commercial complement-competent serum (unknown ABO-type) almost completely lysed WT pRBCs and AB-type hRBCs (possibly ABO-incompatible), and AB-type serum from healthy persons completely lysed WT pRBCs but not AB-type hRBCs (ABO-compatible). However, these sera scarcely lysed genetically modified pRBCs or O-type hRBCs. Meanwhile, owing to the lack of a hemolytic reaction in TKO pRBCs, we could not determine the protective effect of hCD55 expression on TKO pRBCs. Thus, we repeated the experiment using monkey serum containing high titers of anti-porcine cell antibodies. Although 10% serum lysed all pRBCs, regardless of genetic modification, but not O-type hRBCs, 5% monkey serum resulted in lower lysis of TKO/hCD55.hCD39KI and QKO pRBCs compared with that of TKO pRBCs. These results suggest that hCD55 expression partially protects pRBCs from serum-mediated hemolysis. To our knowledge, this is the first evidence that the product of an inserted human gene is functional in pRBCs (6, 35). Because TKO/hCD55.hCD39KI pigs have been developed for solid organ xenotransplantation, hCD39 on pRBCs, which metabolizes ADP and limits platelet activation and aggregation, was not evaluated in this study (6).

It is unclear why QKO pRBCs were better protected in monkey sera than TKO pRBCs. QKO pigs possess *A3GalT2* gene-targeted silencing in addition to TKO genetic modification. The expression of isoglobotriaosylceramide in pRBCs has not yet been reported, and its absence is unlikely to be the reason for QKO pRBC protection. Silencing the *A3GalT2* gene might alter the glycan or protein composition on pRBCs, potentially resulting in the partial protection of QKO pRBCs in sensitized monkey sera. However, it is necessary to compare the lifespans of QKO or TKO pRBCs in the circulation of monkeys before concluding any benefit of QKO modification over TKO modification. Of note, both QKO and TKO pRBCs were well protected from serum-mediated hemolysis and phagocytosis in naïve human sera, comparable to human O-type RBCs, and did not differ with respect to compatibility with naïve human sera. Further comparative analysis of glycan and/or protein profiles as well as *in vivo* studies with TKO and QKO pRBCs would be valuable in predicting the outcomes in the future clinical settings.

To address the extravascular hemolytic reaction, we compared the extent of RBC phagocytosis by THP-1 cells. Phagocytosis by inactivated THP-1 cells is less efficient than when they are activated. However, there is a concern that variable levels of THP-1 cell differentiation during activation might interfere with comparing phagocytosis levels based on the type of RBCs tested. Thus, we used THP-1 cells without any treatment in this study and measured the amount of RBCs taken up by THP-1 cells rather than the percentage of THP-1 cells phagocytosing RBCs. Phagocytosis of RBCs by THP-1 cells was improved by the addition of human serum instead of FBS. In accordance with previous reports (16), WT pRBCs were phagocytosed more than genetically modified pRBCs and O-type hRBCs, with no difference in the extent of phagocytosis. Increased antibody binding to WT pRBCs may explain their facilitated phagocytosis by THP-1 cells. However, the phagocytic activity of RBCs differs depending on the type of phagocytic cells (16, 19). It is important to note that these results may not precisely reflect *in vivo* responses.

As our study was conducted *in vitro*, the findings may not be applicable *in vivo* scenarios since immunological reactions may differ between these conditions. In addition, there are concerns regarding phagocytosis by pRBCs. It is possible that different types of phagocytes in the human body can phagocytose pRBCs in a more facilitated manner than we revealed using THP-1 cells. More importantly, antibodies react to pRBCs *in vivo*, causing antibody-mediated complement activation and phagocytosis and leading to the quick removal of transfused pRBCs (35). Our hypothesis was supported by the hemolysis results obtained using previously sensitized monkey sera containing anti-pig cell antibodies. Therefore, further *in vivo* studies are required to evaluate immunological transfusion reactions and to estimate the lifespan of genetically modified pRBCs in circulation.

Nevertheless, the genetic modification of pRBCs showed significant improvement over WT pRBCs. The immunological reactions of genetically modified pRBCs in naïve human serum did not differ from those of O-type hRBCs in this study, suggesting the potential value of these pRBCs as an RBC substitute in life-threatening emergencies where RBC transfusion is urgently required but not available. However, their cross-matching results should be clarified before transfusion because of individual variations. Our data highlight the improved protection of QKO pRBCs and hCD55 expression on pRBCs with sensitized monkey sera, indicating that there is room for further improving pRBCs for transfusion through the appropriate design of genetic modifications. It is expected that gene modifications, including the insertion of human complement regulatory genes CD55 and CD59, as well as the anti-phagocytosis gene CD47, in TKO or QKO pigs may further improve the survival of pRBCs in the circulation of human and nonhuman primates (35). However, further research is required to confirm this hypothesis.

## Data availability statement

The original contributions presented in the study are included in the article/supplementary material. Further inquiries can be directed to the corresponding author.

## Ethics statement

The studies involving humans were approved by The institutional review board of Hallym University Sacred Heart Hospital. The studies were conducted in accordance with the local legislation and institutional requirements. The human samples used in this study were acquired from a by- product of routine care or industry. Written informed consent for participation was not required from the participants or the participants’ legal guardians/next of kin in accordance with the national legislation and institutional requirements. Ethical approval was not required for the studies on animals in accordance with the local legislation and institutional requirements because only commercially available established cell lines were used.

## Author contributions

SP: Data curation, Visualization, Writing – original draft. HL: Investigation, Writing – original draft. EP: Investigation, Writing – original draft. JR: Conceptualization, Data curation, Funding acquisition, Writing – original draft. PK: Investigation, Writing – original draft. JS: Investigation, Writing – original draft. KC: Conceptualization, Project administration, Writing – original draft. HK: Conceptualization, Data curation, Formal Analysis, Funding acquisition, Methodology, Project administration, Supervision, Validation, Visualization, Writing – original draft, Writing – review & editing.
